# Effect of pilot-scale high-temperature short-time processing on the retention of key micronutrients in a fortified almond-based beverage: implications for fortification of plant-based milk alternatives

**DOI:** 10.3389/fnut.2024.1468828

**Published:** 2024-09-24

**Authors:** Benjamin W. Redan, Joseph Zuklic, Jiarui Cai, Joshua Warren, Coleton Carter, Jason Wan, Amandeep K. Sandhu, Darryl Glenn Black, Lauren S. Jackson

**Affiliations:** ^1^Division of Food Processing Science and Technology, Center for Food Safety and Applied Nutrition, U.S. Food and Drug Administration, Office of Food Safety, Bedford Park, IL, United States; ^2^Institute for Food Safety and Health, Illinois Institute of Technology, Bedford Park, IL, United States

**Keywords:** thermal processing, nutrient degradation, ergocalciferol, elemental analysis, mass spectrometry, minerals, plant-based beverages, vegetarian

## Abstract

The effect of thermal processing treatments on key micronutrients in fortified almond-based beverages has not been well characterized. An almond-based beverage was produced in a pilot plant, fortified with vitamin A palmitate, vitamin D2, riboflavin (vitamin B2), calcium carbonate, and zinc gluconate, and was processed using various high-temperature short-time (HTST) pasteurization treatments. Naturally present micronutrients in the base ingredients included several B vitamins (vitamin B1 [thiamin], total vitamin B3 [sum of nicotinamide and nicotinic acid], and total vitamin B6 [sum of pyridoxal, pyridoxamine, and pyridoxine]) and minerals (magnesium, phosphorus, and potassium). The prepared almond-based beverage was homogenized and thermally processed using HTST pasteurization with a temperature range from ~94 to 116°C for a constant time of 30 s. The samples were analyzed for vitamin A palmitate, vitamin D2, target B vitamins (thiamin, riboflavin, total vitamin B3, and total vitamin B6), and minerals (magnesium, phosphorus, potassium, calcium, and zinc). The results showed that amounts of vitamin A, vitamin D2, riboflavin, and total vitamin B6 did not significantly (*p* > 0.05) change after the HTST treatments, whereas thiamin significantly (*p* < 0.05) decreased by 17.9% after HTST treatment at 116°C. Interestingly, total vitamin B3 content significantly (*p* < 0.05) increased by 35.2% after HTST treatment at 116°C. There was no effect of processing on the minerals that were monitored. The results from this study indicate that the majority of key micronutrients assessed in this study are stable during HTST processing of an almond-based beverage and that fortification of plant-based milk alternatives may be a viable process to enhance the micronutrient content consumers receive from these products.

## 1 Introduction

The increase in consumer demand for plant-based foods that are marketed and sold as alternatives for milk (plant-based milk alternatives or PBMAs) has resulted in a wide assortment of products available in the category (1). PBMAs can currently be broadly classified into those produced using a nut, grain, or legume base. Regardless of whether a PBMA is produced using nuts, grains, or legumes, they can be relatively lower in certain key micronutrients when compared to bovine milk (“milk”) (2–4). Because consumers may use PBMAs as a substitute for milk, some manufacturers fortify these products with micronutrients (e.g., vitamin D, calcium, and vitamin B2 [riboflavin]) to mimic amounts found in fortified dairy products (5, 6) Additionally, it has been reported that consumers prefer PBMAs that are fortified with vitamin A, vitamin D, and calcium (7). Consuming foods, including beverages, containing or fortified with sufficient amounts of key micronutrients is especially critical because many dietary patterns do not result in consumption of the recommended amounts of certain micronutrients, such as calcium and vitamin D (8–10).

High-temperature short-time (HTST) pasteurization is a type of thermal processing that is commonly used by industry for dairy products and other liquid foods to reduce the risk of microbial hazards that may be present in unprocessed foods (11, 12). HTST pasteurization is also used to process PBMAs to produce a product that is stable under refrigerated conditions. Soy-based beverages and other types of PBMAs have been reported to undergo thermal processing at temperatures >80°C to not only inactivate microbial hazards, but also to reduce anti-nutritional factors (e.g., enzyme inhibitors) that may be present in PBMAs (13, 14). Furthermore, beverages fortified with fat-soluble vitamins are also routinely homogenized because, among several reasons, fat-soluble vitamins will partition into the lipid fraction, resulting in potential over- or under fortification of a finished product (12, 14, 15). Another important aspect of processing fortified foods to consider is that key nutrients, such as vitamins A and D, have the potential to degrade under certain processing conditions (16, 17). Previous work has shown that thermal processing of soy-based beverages degrades thiamin (18). Thus, it is critical to have information on the potential degradation of key micronutrients in PBMAs during thermal processing to understand the effect of various processing methods on the nutrient content of these products and to provide a rationale on fortification levels to use for these products.

In this study, a fortified almond-based beverage was developed on a pilot scale to mimic a commercial product. An almond-based beverage was selected for this study because it is one of the most popular types of PBMAs (19). The almond-based beverage was subjected to HTST processing and homogenization to determine the effect of the processing on key micronutrients. Samples were analyzed for vitamin A, target B vitamins, vitamin D2, and for target minerals (magnesium, phosphorus, potassium, calcium, and zinc). The amounts of micronutrients in the processed samples were then compared to the unprocessed sample to determine the retention of these compounds after processing. The results from this study help fill the current gap in knowledge on the effect thermal processing has on key micronutrients in a fortified almond-based beverage.

## 2 Materials and methods

### 2.1 Chemicals, standards, and reference material

Chemicals obtained from Thermo Fisher Scientific (Waltham, MA, USA) were Optima grade acetonitrile, acetone, methanol, formic acid, and concentrated nitric acid; HPLC grade isooctane, methyl-*t*-butyl ether and absolute ethanol; reagent grade 4-phenyl-1,2,4-triazoline-3,5-dione (PTAD), potassium hydroxide, sodium acetate, hydroquinone, papain, glacial acetic acid, hydrochloric acid (36%), pyrogallol, potassium phosphate dibasic, metaphosphoric acid, and retinyl palmitate standard (vitamin A palmitate). Reagent grade acid phosphatase, α-amylase, papain, and ethylenediaminetetraacetic acid disodium salt dihydrate (EDTA) were from Sigma-Aldrich (St. Louis, MO, USA), as well as the standards vitamin D2 (ergocalciferol), *d*6-vitamin D2 (26,26,26,27,27,27-*d*6 ergocalciferol), nicotinamide, pyridoxine hydrochloride, riboflavin, thiamin hydrochloride, pyridoxamine dihydrochloride, and pyridoxal hydrochloride. Nicotinic acid standard was sourced from Toronto Research Chemicals (North York, ON, Canada). Pyridoxamine dihydrochloride standard was purchased from Entegris (Aurora, IL USA). Stable isotopes ^2^H_4_-nicotinamide, ^2^H_4_-nicotinic acid, ^2^H_3_-pyridoxamine, ^13^C_4_-thiamin, and ^13^C_4_,^15^N_2_-riboflavin acid were from Toronto Research Chemicals (North York, ON, Canada). Additionally, ^13^C_4_-pyridoxine and ^2^H_3_-pyridoxal were from Entegris (Aurora, IL USA). Stock standard containing target elements was from Inorganic Ventures (Blacksburg, VA, USA). Internal standard mix for inductively coupled plasma-mass spectrometry (ICP-MS) was purchased from Agilent Technologies (Santa Clara, CA, USA).

Deionized (DI) water (18.2 MΩ cm at 25°C) was obtained from a Milli-Q system (Millipore-Sigma; Burlington, MA, USA). Standard Reference Material (SRM) 1869 (Infant/Adult Nutritional Formula II; milk/whey/soy-based) was from the National Institute of Standards and Technology (NIST; Gaithersburg, MD, USA).

### 2.2 Almond-based beverage formulation

An almond-based beverage was produced in-house using a formulation aimed to mimic the composition of a typical commercially available product (20–22). The almond-based beverage was formulated with approximately 2% weight concentration (weight per weight) (w:w) almond butter, 0.02% (w:w) gellan gum, and 0.2% (w:w) deoiled lecithin powder. The exact amounts of the ingredients are described below in Section 2.3, which describes production. The almond butter (particle size < 254 μm) was from Cache Creek Foods (Woodland, CA, USA). Gellan gum and sunflower deoiled lecithin powder were from Tate & Lyle North America, Inc (Decatur, IL, USA). A vitamin and mineral premix was provided by Caldic North America (Mississauga, ON, Canada). The vitamin and mineral premix is a commercially available product designed to fortify PBMAs and was used in amounts as suggested by the manufacturer. The premix contained calcium carbonate, zinc gluconate, vitamin A (as retinyl palmitate), riboflavin, and vitamin D2 (as ergocalciferol).

### 2.3 Production of the almond-based beverage

**Figure 1:**
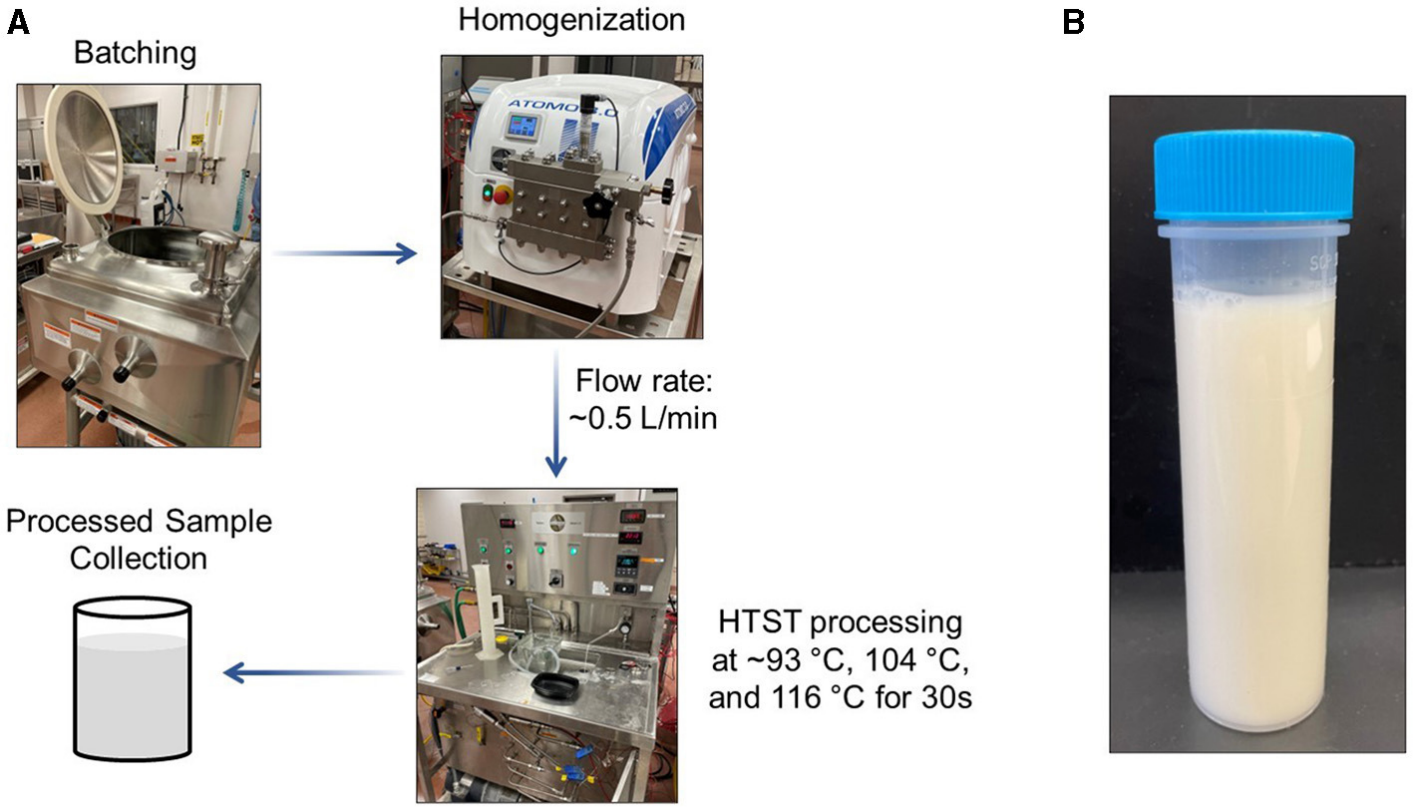
**(A)** Schematic of the processing design for pilot scale production of an almond-based beverage; **(B)** Representative image of the finished almond-based beverage. HTST, high-temperature short-time.

The almond-based beverage was produced and processed over three independent trials in the pilot plant facility at the Institute for Food Safety and Health (IFSH; Bedford Park, IL, USA). An overview of the experimental process is shown in Figure 1A. Each batch size was approximately 75 L. First, 75.0 kg of tap water was placed into a Breddo Likwifier mixer (Ensight Solutions, LLC; Strafford, MO, USA). Almond butter (1,503.1 ± 1.9 g), 15.0 ± 0.0 g of gellan gum, and 150.0 ± 0.1 g of lecithin were added into the water and mixed for 15 min at ambient temperature. Afterwards, 253.2 ± 0.1 g of vitamin/mineral premix was added and mixed for an additional 15 min at ambient temperature. After mixing, 1 L of the almond-based beverage was sampled from the mixer for micronutrient analysis (designated as the unprocessed sample). The mixing was continued throughout the production time period.

The almond-based beverage was then processed by pumping it through an Atomo 3.0 homogenizer (Bertoli; Reggio Emilia, Italy) followed by a Bantam 1-S HTST Pasteurizer (MicroThermics; Raleigh, NC, USA). Processing parameters were selected based on pasteurization conditions used for PBMAs (14, 23). The selected conditions would be expected to eliminate greater than 5 log of vegetative pathogens and most spoilage organisms (24). The homogenizer unit was set to 179 bar for all thermal treatment conditions. Because of the importance of monitoring temperature during the experiment (25), temperatures were recorded with a thermocouple. After homogenization, the almond-based beverage underwent thermal treatment at 93.7 ± 0.2°C, 105.2 ± 0.2°C, and 116.3 ± 0.2°C. The flow rate was 508.3 ± 2.9 mL/min with a 30 s hold time in the pasteurizer. The almond-based beverage was rapidly cooled using a water supply line containing ambient temperature water. For all temperature treatments, the almond-based beverage was 29.9 ± 1.0°C at the heater intake and 26.9 ± 1.9°C at the cooler outlet. A sample of the homogenized and pasteurized almond-based beverage was collected at the pasteurizer outlet in a Nalgene bottle. The finished product is shown in Figure 1B. Three independent trials were performed at each pasteurization temperature. For the second and third trials, a “homogenized only” sample was produced that was homogenized at relatively low temperatures (< 46.1°C) for use as the control for vitamin A data only. All collected samples were stored at −20°C until analysis.

### 2.4 Vitamin A analysis using HPLC-DAD

The samples were analyzed for vitamin A as retinyl palmitate using AOAC Official Method 2012.10 (26). In brief, after the frozen samples were thawed and thoroughly mixed, 5.0 g of each sample was dispensed into an amber 50 mL polypropylene tube (Chemglass Life Sciences; Vineland, NJ, USA). This was followed by the addition of 5 mL of enzymatic digestion solution (papain:sodium acetate:hydroquinone; 20 g/L:40 g/L:1.0 g/L). Samples were incubated in a 37°C water bath for 25 min. After the samples were cooled to room temperature, 20 mL of acidified methanol (20 mL acetic acid + 1 L methanol) was added to each sample and then samples were shaken for 10 min using a GenoGrinder automated shaker (SPEX SamplePrep; Metuchen, NJ, USA). Each sample was mixed with iso-octane (10 mL) and then placed in the shaker for an additional 10 min. The samples were centrifuged (Sorvall Legend X1R; Thermo Fisher Scientific, Waltham, MA, USA), and approximately 1 mL of the organic layer from each sample was transferred to an amber LC vial (Waters Co., Bedford, MA, USA) for analysis. An amber vial was used to prevent degradation of the light-sensitive micronutrients.

Retinyl palmitate was quantified using high-performance liquid chromatography (HPLC; Agilent 1260 Infinity; Santa Clara, CA, USA) equipped with a photodiode array detector (HPLC-DAD). Separations were achieved using a Zorbax NH2 column (150 × 4.6 mm, 5 μm; Agilent Technologies, Santa Clara, CA, USA). A linear gradient elution was used with mobile phase A as *n*-hexane, and mobile phase B as *n*-hexane:methyl-*t*-butyl ether:methanol (750:250:3). The flow rate was set to 1.5 mL/min, and the total elution cycle was 20 min. Sample injection volume was 50 μL, and both *cis*- and *trans*-retinyl palmitate isomers were monitored at 325 nm. Quality control measures for analysis included validating each analytical sequence by analyzing a sample of SRM 1869.

### 2.5 Vitamin D2 analysis using UPLC-MS/MS

Vitamin D2 analysis was completed using AOAC First Action Method 2016.05 (27). All steps during the extraction were performed under UV-shielded lighting. In brief, after the frozen samples were thawed and thoroughly mixed, 10 mL of each sample was dispensed into an amber 50 mL polypropylene tube (Chemglass Life Sciences; Vineland, NJ, USA), followed by addition of 10 mL ethanolic pyrogallol solution (1%, mass concentration (weight per volume) (w:v), 0.5 mL of internal standard solution containing 1.0 μg/mL *d*6-vitamin D2 and 2 mL of 50% potassium hydroxide solution (w:v). Samples were saponified for 1 h, and lipid-soluble compounds were extracted using isooctane. Vitamin D2 was derivatized with 75 μL PTAD solution (10 mg/mL). The derivatized sample was mixed with acetonitrile (1 mL), and then centrifuged. The lower layer was collected and mixed with 167 μL DI water. The filtered sample was dispensed into an LC-MS vial (Waters Co., Bedford, MA, USA) for analysis.

Vitamin D2 was quantified using a ultraperformance liquid chromatography (UPLC; Acquity; Waters Co., Bedford, MA, USA) UPLC in line with a TQD triple quadrupole mass spectrometer (UPLC-MS/MS). Separations were achieved using a CORTECS C18 core-shell column (2.1 × 50 mm, 2.6 μm; Waters Co., Bedford, MA, USA). Gradient elution mode was used with aqueous 0.1% volume concentration (volume per volume) (v:v) formic acid as mobile phase A and methanol as mobile phase B. The flow rate was set to 0.6 mL/min, and the elution cycle was 5.5 min. Sample injection volume was 3 μL. The TQD mass spectrometer used an electrospray ionization (ESI) probe operated in positive ionization mode. The mass transition for vitamin D2 quantification was mass-to-charge ratio (*m/z*) 572.2 → 298.0. For the internal standard, the mass transition for *d*6-vitamin D2 quantification was *m/z* 578.2 → 298.0. Quality control measures for analysis included validating each analytical sequence by analyzing a sample of SRM 1869.

### 2.6 Vitamin B complex analysis using UHPLC-MS/MS

Analysis of target B vitamins (thiamin, riboflavin, total vitamin B3, total vitamin B6) was completed using AOAC Method 2015.14 (28). In brief, 1.0 g of sample plus 100 μL of internal standard mixture (containing ^2^H_4_-nicotinamide, ^2^H_4_-nicotinic acid, ^13^C_4_-pyridoxine stock, ^2^H_3_-pyridoxal, ^2^H_3_-pyridoxamine, ^13^C_4_-thiamine stock, and ^13^C_4_,^15^N_2_-riboflavin acid) was added to a 50 mL centrifuge tube and quickly mixed using a vortex. An enzyme cocktail (5 mL) consisting of 1 mg/mL acid phosphatase and 1 mg/mL papain in 50 mM ammonium formate buffer (pH 4.0) was added to the centrifuge tube, which was then placed in a shaking water bath overnight at 37°C. After the sample was incubated, 50 mM ammonium formate was added to dilute the sample to approximately 30 mL. The sample was filtered through a 0.45 μm PTFE syringe filter, and further diluted ~17-fold with 50 millimolar (mM) ammonium formate before being transferred to an HPLC vial for analysis.

Target B vitamins were quantified using ultrahigh-performance liquid chromatography (UHPLC; Agilent model 1260 Infinity I; Santa Clara, CA, USA) in line with a 6460 series triple quadrupole mass spectrometer (UHPLC-MS/MS; Agilent Technologies, Santa Clara, CA, USA) with an ESI probe operated in positive ionization mode. Separations were achieved using a Waters Acquity BEH C18 column (2.1 × 100 mm, 1.7 μm; Waters Co., Bedford, MA, USA). Gradient elution mode was used with 20 mM ammonium formate in water as mobile phase A and methanol as mobile phase B. The flow rate was set to 0.35 mL/min. Quantification of the B vitamin compounds was conducted using multiple-reaction monitoring (MRM) mass transitions. Optimization of collision energies, fragmentor voltages, and MRM mass transitions was done using the Mass Hunter Optimizer. MRM mass transitions were used as reported by McClure (28). Quality control measures for analysis included validating each analytical worklist by analyzing a sample of SRM 1869.

Total vitamin B3 is reported as the sum of nicotinamide and nicotinic acid. Total vitamin B6 is the sum of pyridoxal, pyridoxamine, and pyridoxine. Thiamine and riboflavin are reported as the single measured form.

### 2.7 Mineral analysis using ICP-MS

Samples were analyzed for total magnesium (Mg), phosphorus (P), potassium (K), calcium (Ca), and zinc (Zn) content using microwave-assisted digestion followed by ICP-MS, according to a method adapted from the U.S. Food and Drug Administration's (FDA's) Elemental Analysis Manual 4.7 (29). In brief, 2.0 g of sample was placed in a digestion vessel with 5 mL of nitric acid and subjected to microwave-assisted digestion using a Discover SP-D microwave digestion system (CEM Corporation; Matthew, NC, USA). After digestion, each sample was diluted to 50 g with DI water in trace metal quality plastic tubes (SCP Sciences; Champlain, NY). Samples were further diluted approximately 25-fold with 5% (v:v) aqueous nitric acid, except for samples used for Zn analysis, which were not diluted. An Agilent 8800 ICP-MS (Santa Clara, CA, USA) was set in single quadrupole mode with research grade helium (99.999%) from Airgas (Radnor, PA) as the collision gas. Internal Standard Mix (Agilent Technologies; Santa Clara, CA, USA) was prepared in 5% aqueous nitric acid (v:v). The internal standard was set to scandium (^45^Sc) for Mg, K, and Ca; germanium (^72^Ge) for P; and rhodium (^103^Rh) for Zn. Data for total Mg, P, K, Ca, and Zn are reported as ^24^Mg, ^31^P, ^39^K, ^66^Zn, and ^44^Ca, respectively. Quality control measures for analysis included validating each analytical sequence by analyzing SRM 1869, in addition to analyzing a continuing calibration verification standard every 10 samples.

### 2.8 Data processing and statistics

Vitamin A data were processed using ChemStation version C.01.05 (Agilent Technologies; Santa Clara, CA, USA). Vitamin A results are expressed as retinol activity equivalents (RAE), which is calculated by multiplying the results for retinyl palmitate (the sum of *cis* and *trans* isomers) by 0.55 (26). B vitamin data were processed using MassHunter Workstation software version B.08 (Agilent Technologies; Santa Clara, CA, USA). Data processing for vitamin D results was conducted using MassLynx workstation software version 4.1 (Waters Co.; Bedford, MA, USA). Mineral data processing was conducted using MassHunter Workstation software version 4.6 (Agilent Technologies; Santa Clara, CA, USA) with results exported as Excel worksheets (Microsoft Office, Microsoft Co., Redmond, WA).

Two subsamples from each experimental trial underwent extraction for vitamin analyses, and three subsamples for mineral analysis. Statistical analysis was performed using JMP 17 (SAS Institute, Cary, NC, USA). One-way analysis of variance (ANOVA) followed by pairwise mean comparisons using Tukey's honest significant difference (HSD) *post-hoc* correction determined significant differences (*p* < 0.05) between treatments. Data for figures are displayed as means ± SD.

## 3 Results

### 3.1 Overview of the micronutrient content of the almond-based beverage

Table 1 displays a summary of the fortified and naturally occurring micronutrients in the almond-based beverage. We were able to measure the fortified vitamins (vitamin A, vitamin D2, riboflavin) and minerals (calcium and zinc) in both the unprocessed sample and sample that was homogenized and then processed using the HTST pasteurizer. In addition to the fortified micronutrients, we were also able to measure naturally occurring micronutrients from the base ingredients that included target B vitamins (thiamin, total vitamin B3, and total vitamin B6) and minerals (magnesium, phosphorus, and potassium). Percent recovery of the fortified micronutrients (except for vitamin A) in the unprocessed almond-based beverage ranged from 84% for riboflavin to 96% for calcium and zinc. Initial analysis showed variability in vitamin A levels between the unprocessed sample and sample that underwent homogenization. Thus, we used homogenized-only samples (thermal exposure was < 46.1°C for these samples) as the control for processing experiments for the vitamin A data only.

**Table 1:** Summary of the fortified and naturally-occurring micronutrients in the almond-based beverage produced in a pilot plant.

**Micronutrient**	**Fortified or naturally occurring**	**Amount of micronutrient added/100 g beverage**	**Measured amount (mean±SD) in 100 g unprocessed beverage^b^**	**Average Recovery (%)**
Vitamin A	Fortified	47.5 μg RAE^a^	44.7 ± 6.1 μg RAE	94
Thiamin (vitamin B1)	Naturally occurring	-	2.0 ± 0.2 μg	-
Riboflavin (vitamin B2)	Fortified	172.5 μg	145.0 ± 2.3 μg	84
Total vitamin B3^c^	Naturally occurring	-	3.4 ± 0.1 μg	-
Total vitamin B6^c^	Naturally occurring	-	44.5 ± 10.7 μg	-
Vitamin D2	Fortified	1.0 μg	0.89 ± 0.03 μg	90
Magnesium	Naturally occurring	-	7.7 ± 0.3 mg	-
Phosphorus	Naturally occurring	-	15.1 ± 1.8 mg	-
Potassium	Naturally occurring	-	15.3 ± 0.5 mg	-
Calcium	Fortified	131.3 mg	125.8 ± 14.0 mg	96
Zinc	Fortified	420.0 μg	403.1 ± 53.9 μg	96

^a^RAE, retinol activity equivalents.

^b^Unprocessed sample is almond-based beverage that was mixed but did not undergo high temperature-short time (HTST) processing and homogenizaton.

^c^Total vitamin B3 is the sum of nicotinamide and nicotinic acid. Total vitamin B6 is the sum of pyridoxal, pyridoxamine, and pyridoxine.

### 3.2 Retention of fat-soluble vitamins A and D during processing of an almond-based beverage

**Figure 2:**
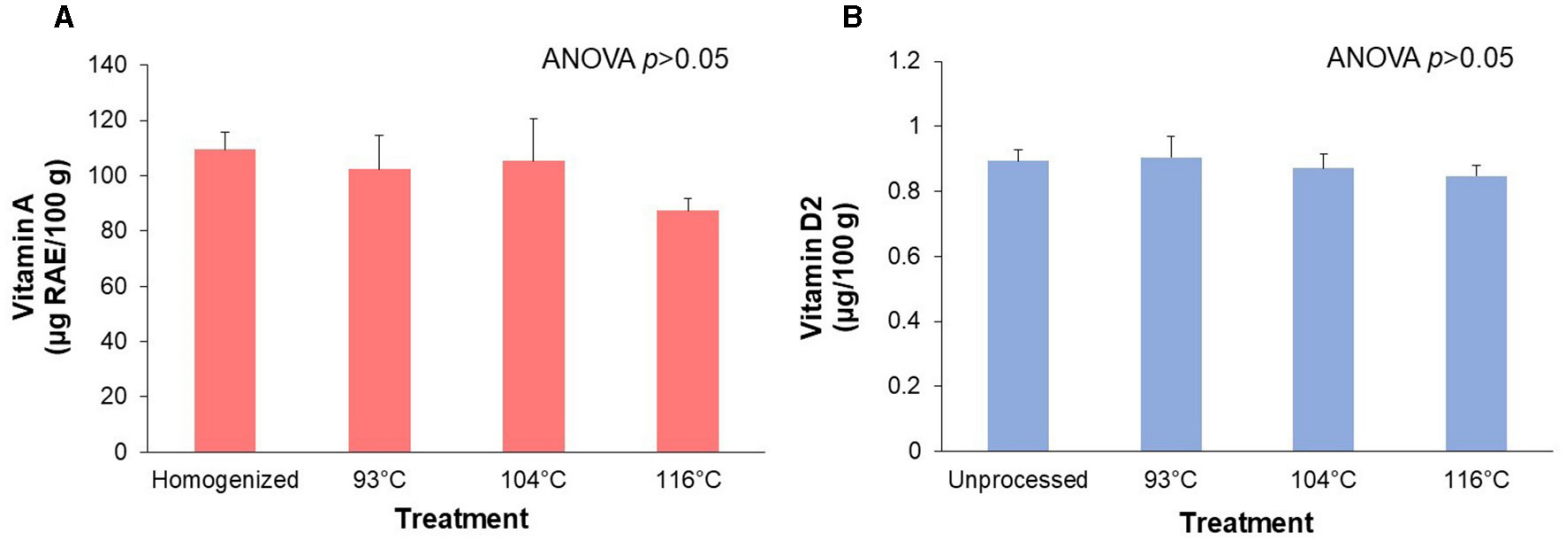
Retention of the fat-soluble micronutrients after processing of an almond-based beverage. **(A)** Retention of vitamin A and **(B)** vitamin D2 after different high temperature-short time (HTST) processing treatments. The almond-based beverage was fortified with vitamin A (as retinyl palmitate) and vitamin D2 (ergocalciferol). ANOVA indicated that there was no significant effect of temperature treatments on vitamin A or vitamin D amounts. Values are shown as means±SD of three trials, except for the homogenized sample for vitamin A (*n* = 2).

Figure 2 depicts the retention of vitamin A palmitate (Figure 2A) and vitamin D2 (Figure 2B) over three processing temperatures (~93°C, 104°C, and 116°C). There was no significant (ANOVA *p* > 0.05) effect of HTST processing temperature on either vitamin A palmitate or vitamin D2 content. Although not statistically significant, the almond-based beverage processed at the highest temperature treatment (116°C) was 20.3% lower in vitamin A palmitate compared to the homogenized control. We also found no significant (*p*>0.05) differences in amounts of *cis* or *trans* vitamin A palmitate isomers across treatments (data not shown). The almond-based beverage processed at the highest temperature treatment (116°C) was 5.2% lower in vitamin D2 compared to the unprocessed control.

### 3.3 Retention of target B vitamins (thiamin, riboflavin, vitamin B3, and vitamin B6) during processing of an almond-based beverage

**Figure 3:**
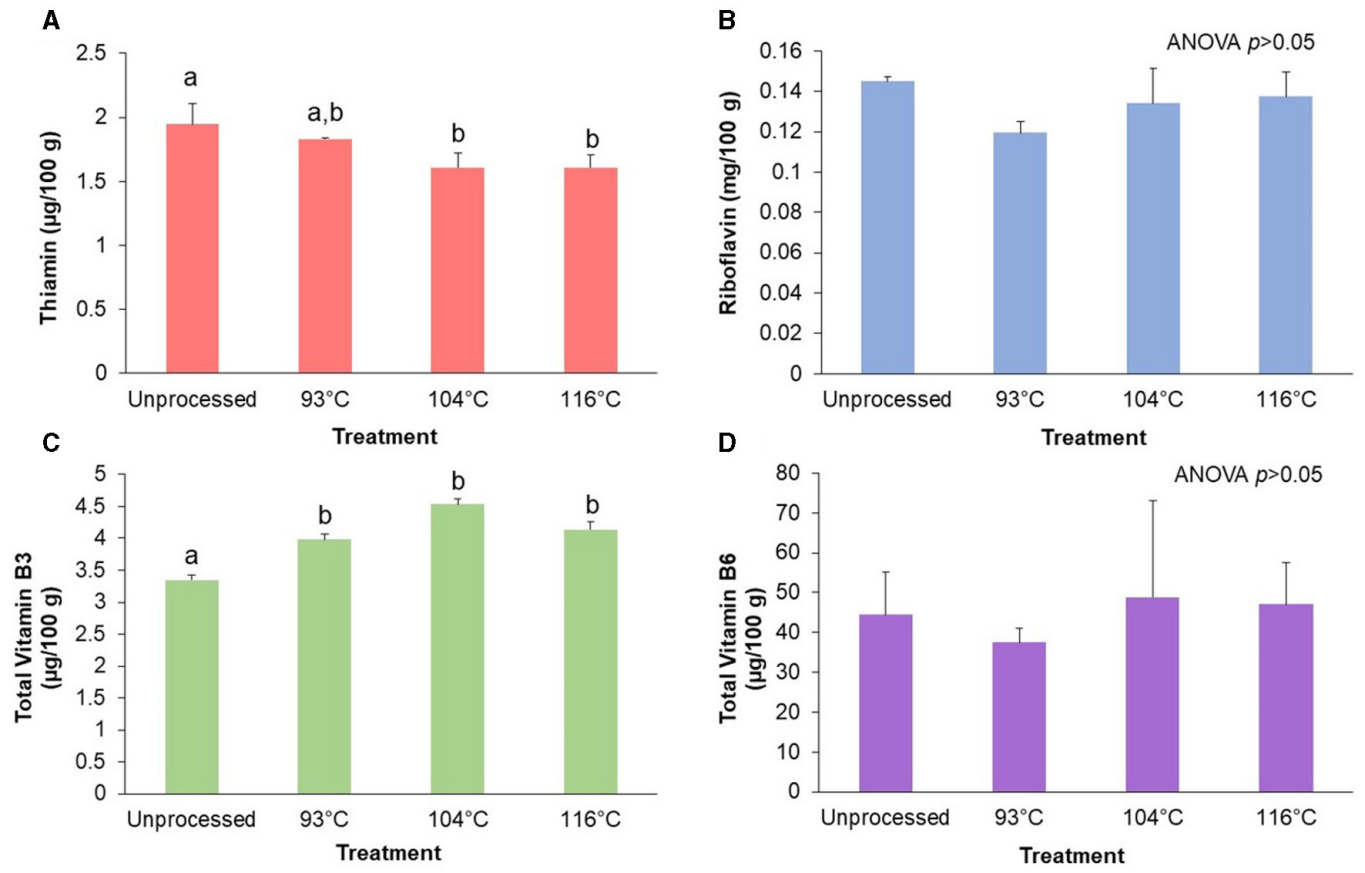
Retention of target B vitamins after processing of an almond-based beverage. Retention of **(A)** thiamin, **(B)** riboflavin, **(C)** total vitamin B3, and **(D)** total vitamin B6 after different high temperature-short time (HTST) processing treatments. The almond-based beverage was fortified with riboflavin; other B vitamins (thiamin, total vitamin B3, and total vitamin B6) were naturally occurring in the base ingredients. Total vitamin B3 is the sum of nicotinamide and nicotinic acid. Total vitamin B6 is the sum of pyridoxal, pyridoxamine, and pyridoxine. Thiamin and riboflavin are reported as the single measured form. Temperature treatments not sharing the same letter within a B vitamin are significantly different (*p* < 0.05) as determined by the Tukey HSD test. Values are shown as means ± SD of three trials.

Figure 3 shows the effect of HTST processing the almond-based beverage on the B vitamins thiamin, riboflavin, total vitamin B3, and total vitamin B6. The results indicate that there was a significant effect (*p* < 0.05) of processing temperature on thiamin content (Figure 3A). Pairwise comparisons showed a clear inverse relationship between increasing temperature and the thiamin content. Processing of the almond-based beverage at the highest temperature (116°C) resulted in 17.9% less thiamin (*p* < 0.05) compared to the unprocessed almond-based beverage. In contrast, both riboflavin (Figure 3B) and total vitamin B6 (Figure 3D) were not significantly (*p*>0.05) affected by the processing temperature. The unprocessed control differed by 6% in riboflavin and total vitamin B6 content compared to the highest temperature treatment.

There was a significant (*p* < 0.05) increase in amounts of total vitamin B3 after processing (Figure 3C). Pairwise comparisons showed that all processing temperatures had significantly (*p* < 0.05) higher amounts of total vitamin B3 compared to the unprocessed control. The total vitamin B3 amounts increased in the almond-based beverage from 18.8% to 35.2% after processing, compared to the unprocessed control.

### 3.4 Retention of target minerals during processing of an almond-based beverage

**Figure 4:**
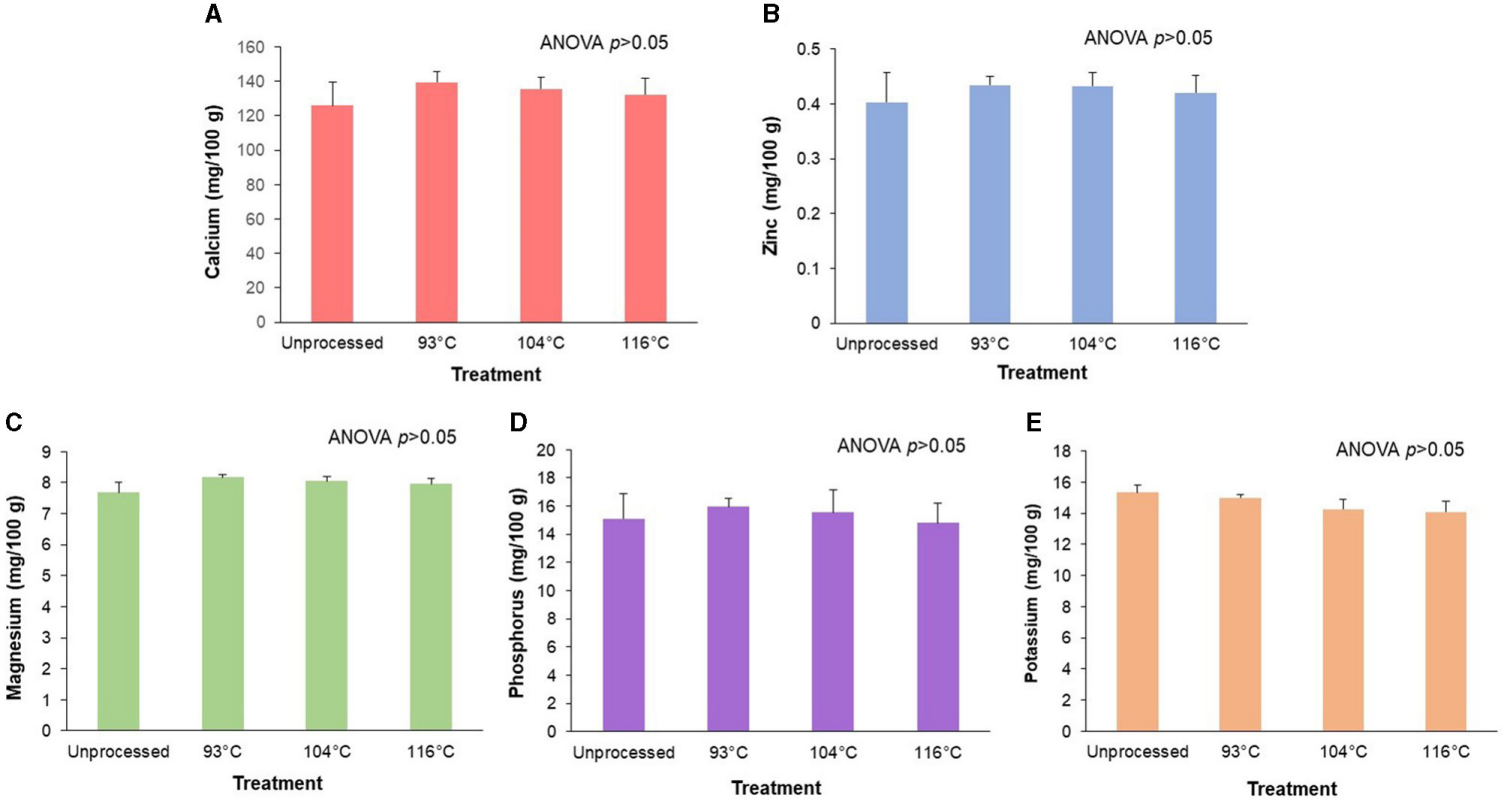
Retention of target minerals after processing of an almond-based beverage. Retention of **(A)** calcium, **(B)** zinc, **(C)** magnesium, **(D)** phosphorus, and **(E)** potassium after different high temperature-short time (HTST) processing treatments. The almond-based beverage was fortified with calcium carbonate and zinc gluconate; magnesium, phosphorus, and potassium are naturally occurring minerals from the base ingredients. Data for total magnesium, phosphorus, potassium, calcium, and zinc are reported as ^24^Mg, ^31^P, ^39^K, ^44^Ca, and ^66^Zn, respectively. ANOVA indicated that there was no significant effect of temperature treatments on the amount of any target mineral. Values are shown as means ± SD of three trials.

Processing did not have a significant effect (*p* > 0.05) on either the amounts of the fortified minerals (calcium and zinc) or the naturally occurring minerals (magnesium, phosphorus, and potassium) (see Figure 4). Although it did not reach statistical significance, there was a trend (*p* = 0.06) for an effect of processing on potassium amounts, with an 8% decrease at the highest temperature treatment compared to control (Figure 4E).

## 4 Discussion

Foods and beverages fortified with key micronutrients are an important part of the modern diet because they can supply critical micronutrients that would not otherwise be obtained in sufficient amounts through the diet (30). Because of the large increase in the consumption of PBMAs, there have been efforts to establish guidelines for fortification of these products with key micronutrients (31, 32). One important aspect of micronutrient fortification that needs to be considered is that certain vitamins can be susceptible to degradation from various factors, such as thermal exposure, pH, oxygen, and UV radiation (16, 33). These factors affect each vitamin differently due to dissimilarities in their chemical structures. In this study, we aimed to investigate the effect of thermal processing on certain micronutrients on a pilot scale in order to mimic the commercial production process of an almond-based beverage.

Manufacturers will usually add an “overage” of a micronutrient to compensate for degradation of a fortified micronutrient during processing and storage (33). For milk undergoing pasteurization or ultra-high temperature (UHT) processing, an overage of 20–30% for vitamins A and D, and 15–50% for B vitamins have been recommended (33). Thiamin is very sensitive to thermal exposure and other factors (34), so an overage of 50% above the label claim has been recommended for UHT milk. A smaller percent overage for minerals (~5%) has been recommended to compensate for effects such as loss from precipitation or reaction with food components (33). This overage helps to ensure compliance with governmental regulations—such as those from the Canadian Food Inspection Agency and the FDA—which require that the content of a composite sample of a product be at least the value declared on the product's nutrition information labeling (35, 36). Although the degradation of key micronutrients in dairy products has been extensively studied, only a limited amount of published data exists on the effect of thermal processing on micronutrients in PBMAs (17, 37).

Our findings overall indicate that, under the HTST conditions studied, levels of measured micronutrients were largely not affected by processing the almond-based beverage. We observed no significant reduction in vitamin A palmitate after HTST processing. Although vitamin A palmitate is not highly susceptible to degradation from thermal processing (15, 38), vitamin A palmitate can undergo *cis*-*trans* isomerization from certain heat treatments (39). Isomerization of vitamin A is significant from a nutritional standpoint because the major products of all-*trans* retinyl palmitate isomerization (13-*cis* and 9-*cis* isomers) have reduced biological activity. Our data did not find that such isomerization occurred to any significant extent. Experiments by Schwartz (39) reported canning milk at 117°C for 20 min did not result in isomerization of all-*trans* retinyl palmitate. Considering that the most severe processing conditions in our experiments were approximately 116°C for 30 s, it is not surprising that we did not observe significant isomerization.

As with vitamin A palmitate, there was no significant reduction of vitamin D2 in the almond-based beverage after thermal processing. Previous research on degradation of vitamin D2 in milk reported that pasteurization at a lower temperature but for a longer time (63°C for 30 min) than our study did not reduce amounts of vitamin D2 (17). Similar to vitamin A, vitamin D2 does not appear to be highly susceptible to degradation from thermal exposure (17).

Because of its known susceptibility to degradation, thiamin has been the topic of various studies to identify conditions that enhance its stability in foods (13, 34, 40). Accordingly, our results showed that thiamin was the only micronutrient that was significantly lower after all processing conditions, decreasing 17.9% at the highest processing temperature (116°C). Kwok et al. (18) found that thermal treatment of a soy-based beverage at 120°C for 30 s resulted in a thiamin loss of 32%, which is comparable to our results in the almond-based beverage.

Both riboflavin and vitamin B6 were not affected by the thermal treatments under the experimental conditions. This aligns with previous work done in milk and soy-based beverages (13, 18, 41). Interestingly, we found that there was up to a 35.2% increase in the amount of vitamin B3 after thermal processing. It is plausible that the increase may be due to the release of bound niacin from the almonds (42, 43). Additionally, the changes may be due to metabolic intermediates with a niacin moiety converting to vitamin B3 during the thermal treatment, which can occur when processing grains (44).

Minerals in certain PBMAs on the market have been reported to form insoluble complexes and precipitate (45). Because oxalates (present in almonds) can form an insoluble complex with minerals (46), we expected that there would be a decrease in target minerals after processing due to the formation of these insoluble complexes. Our results, however, showed that there was no significant effect of processing on the amounts of the fortified minerals (calcium and zinc) or the naturally occurring minerals from the base ingredients (magnesium, phosphorus, and potassium) of our almond-based beverage. This indicates that the conditions used in our study prevent significant precipitation of the minerals during batching of the ingredients and processing.

There are a few limitations of our study that we should note. We aimed to investigate the effects of thermal processing on the target micronutrients, but there are other factors, such as the shelf life of the product, that need to be considered when determining an overage amount for a particular micronutrient that should be added to meet a label declaration or claim. We used HTST processing for our experiments, but UHT processing is often used to process PBMAs to produce a shelf-stable product. Our experimental results may differ from processing that uses UHT treatment because this type of processing generally occurs at temperatures above 135°C, compared to a maximum of 116°C used in our study (47). We performed our study using a nut-based matrix (almond), but there may be different effects on micronutrients when thermally processing a PBMA produced from grains (e.g., oat-based beverages), legumes (e.g., pea protein-based beverages), or other plant material due to differences in the composition of these beverages compared to those made from almond. Finally, we note that we did not assess whether processing affected micronutrient bioavailability. Thermal processing of plant-based foods, including PBMA, is known to potentially enhance the bioavailability of macronutrient and micronutrients by reducing antinutritional factors and should be considered in future work (13).

## 5 Conclusion

We performed homogenization and HTST processing on an almond-based beverage at a pilot-scale level to determine the potential effects of processing on the amounts of key micronutrients. There was no significant degradation of the fortified micronutrients in the beverage, including vitamin A, vitamin D2, riboflavin, and total vitamin B6. There was some degradation of naturally occurring thiamin, but, interestingly, there was a small increase in total vitamin B3 amounts. There was no change in the amounts of either the fortified minerals (calcium and zinc) or the naturally occurring minerals (magnesium, phosphorus, and potassium). These data suggest that most key micronutrients do not require a high overage to compensate for degradation occurring from HTST processing used to produce an almond-based beverage.

This study provides important information on the effect of HTST treatments on key micronutrients in an almond-based beverage. Because of the current popularity of almond-based beverages, our data are of significant interest to a large segment of the PBMA market. Our described experimental design can be modified for future studies to assess additional PBMA types, such as those made from grains, legumes, or other plant material. These results can help inform best practices for fortification of PBMAs processed using HTST treatments to ensure that consumers obtain a product with micronutrients that meet a label declaration or claim.

## Data Availability

The original contributions presented in the study are included in the article/supplementary material, further inquiries can be directed to the corresponding author.
